# A combined RAD-Seq and WGS approach reveals the genomic basis of yellow color variation in bumble bee *Bombus terrestris*

**DOI:** 10.1038/s41598-021-87194-y

**Published:** 2021-04-12

**Authors:** Sarthok Rasique Rahman, Jonathan Cnaani, Lisa N. Kinch, Nick V. Grishin, Heather M. Hines

**Affiliations:** 1 Department of Biology, The Pennsylvania State University, 208 Mueller Labs, University Park, PA USA; 2 Department of Biological Sciences, The University of Alabama, Tuscaloosa, AL 35487 USA; 3 AgroBee, 8682000 Ein-Yahav, Israel; 4 Howard Hughes Medical Institute and Departments of Biophysics and Biochemistry, University of Texas Southwestern Medical Center, Dallas, TX USA; 5 Department of Entomology, The Pennsylvania State University, University Park, PA USA

**Keywords:** Genetics, Genomics, Evolution, Evolutionary genetics, Social evolution

## Abstract

Bumble bees exhibit exceptional diversity in their segmental body coloration largely as a result of mimicry. In this study we sought to discover genes involved in this variation through studying a lab-generated mutant in bumble bee *Bombus terrestris,* in which the typical black coloration of the pleuron, scutellum, and first metasomal tergite is replaced by yellow, a color variant also found in sister lineages to *B. terrestris*. Utilizing a combination of RAD-Seq and whole-genome re-sequencing, we localized the color-generating variant to a single SNP in the protein-coding sequence of transcription factor *cut*. This mutation generates an amino acid change that modifies the conformation of a coiled-coil structure outside DNA-binding domains. We found that all sequenced Hymenoptera, including sister lineages, possess the non-mutant allele, indicating different mechanisms are involved in the same color transition in nature. *Cut* is important for multiple facets of development, yet this mutation generated no noticeable external phenotypic effects outside of setal characteristics. Reproductive capacity was reduced, however, as queens were less likely to mate and produce female offspring, exhibiting behavior similar to that of workers. Our research implicates a novel developmental player in pigmentation, and potentially caste, thus contributing to a better understanding of the evolution of diversity in both of these processes.

## Introduction

Understanding the genetic architecture underlying phenotypic diversification has been a long-standing goal of evolutionary biology. In early research, discoveries and understanding of the genetic basis of traits relied on fortuitous mutant phenotypes predominantly in *Drosophila*^1,2^ and subsequently in other model organisms (reviewed in Ref.^3^). These studies have not only contributed myriad insights about the characteristics, chromosomal arrangement, and functional interactions of involved genes but have also shed light on the complex genomic mechanisms involved in natural variation^4^. Many of these early-era forward genetics studies^5^ involved color-variable mutants. Such color variants have led the way in our understanding of evolutionary genetic processes, as coloration tends to be under strong selection and thus exhibits substantial and sometimes complex variation^6^.

In recent years, the emergence of increasingly cheaper high-throughput sequencing techniques and availability of genomic resources and computational tools have expanded investigation of the genomic basis of color traits to a wide range of non-model organisms. Application of high-throughput sequencing (e.g., Whole Genome Sequencing, RNA-Seq, RAD-Seq) has provided solutions to many practical complications (e.g., knowledge gap in trait heritability, insufficient pedigree data, the infeasibility of lab-rearing or crossing) that previously limited the ability to unravel the genomic basis of color traits in many non-model organisms^7^. While some of this research has identified genes which have recurrent pigment-related roles in model organisms, many of these studies have provided novel insights about the genetic targets driving pigment variation (reviewed in Ref.^6,8^). Such research has also contributed to broad principles in evolutionary genetics through revealing the multiple genomic routes (e.g., acting in *cis* and *trans*) to color variation (reviewed in Ref.^6,9,10^) in both model and non-model organisms. These studies have also revealed the importance of co-option of major developmental genes for color patterning (e.g., Ref.^11,12^), and the role of linked genetic variants in facilitating complex mimetic color phenotypes (e.g., Ref.^13,14^).

Bumble bees exhibit an astounding diversity of color patterns, with the ~ 260 species^15^ of this genus displaying > 400 color patterns^16,17^. This striking diversity has been largely attributed to the repeated divergence and convergence in color patterns onto numerous local Müllerian mimicry complexes, however, other ecological factors, such as thermoregulation and crypsis, may also be involved^16^. Color is imparted in the thick setal pile (pubescence) on the head, thorax and abdominal sclerites in these bees, and is highly modular, with transitions between several colors (e.g., black, red, white, orange, yellow) across segments and many of the possible conceivable segmental combinations of these colors occurring across the lineage^17^. This phenotypically diverse genus is an emerging model system in evolutionary research^7^, as this system contains ample polymorphisms that enable discovery of the genetic basis of coloration in natural populations^12,18^, can reveal evo-devo processes in segmental modularity^12^, and can disentangle the microevolutionary processes involved in sorting allelic variation through its abundant natural replicates of identical segmental color transitions^7,12^.

*Bombus terrestris* is one of the most abundant bumble bee species in temperate regions of the western Palearctic. It is a major commercially reared pollinator utilized globally for greenhouse pollination services^19^, a development which has facilitated its use in laboratory studies. *B. terrestris* has become the leading model bumble bee for research in such areas as social evolution^20^, learning^21^, foraging behavior^22^, immunology^23^, ecological and landscape genetics^24^, impacts of anthropomorphic threats (e.g., pesticides, pathogens)^25^, flight^26^ and thermoregulation^27^. In recent years, research on *B. terrestris* has been facilitated by the availability of multifaceted genomic resources as it has a publicly available annotated genome^28^ and numerous additional available genomic^29^ and transcriptomic^30^ datasets.

**Figure 1:**
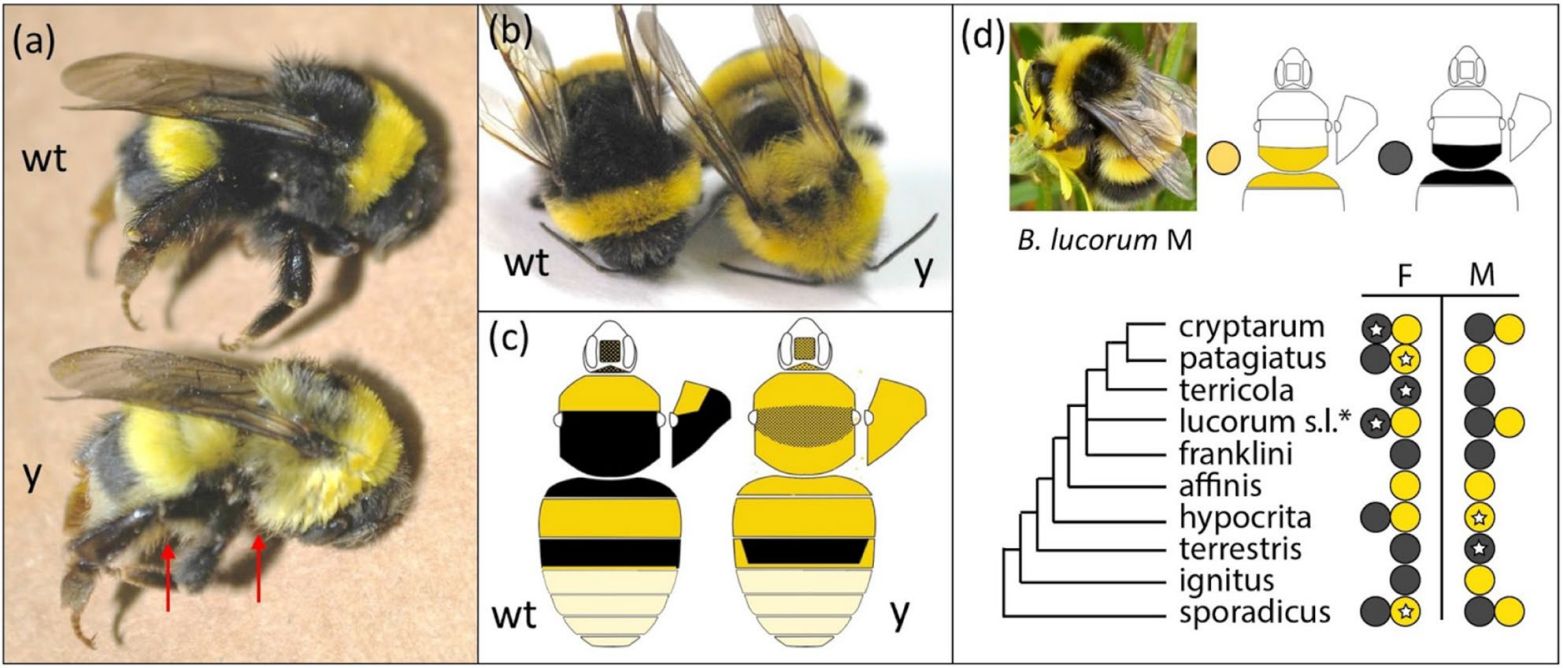
Wildtype (wt) and Yellow (y) mutant color variants of *B. terrestris*. (**a**) Lateral view of wt and y workers. Red arrows point to yellow leg setae and ventral setae. (**b**) Dorsal view of workers. (**c**) Diagram of coloration pattern differences found in both morphs, showing the pattern for males, which matches the patterns for females. Stipules indicate mixed color. (**d**) The incidence of this phenotype (black vs. yellow patterns on the third thoracic plus first metasomal segment) in the *Bombus s.s.* clade containing *B. terrestris*. Some species are polymorphic in this color and species vary in whether this color is sex-specific [male (M), female (F)]. White stars in circles indicate specimens sampled for this study. Intermediates with mixing of setal colors in these segments can sometimes be found. *B. lucorum s.l.* includes a complex of species, to which color assignment has not been ascribed^31^. The inset photograph (cropped from Ref.^92^, creative commons license) depicts a yellow *B. lucorum* male morph from Scotland, which displays the same color pattern as the mutant *B. terrestris* phenotype. Phylogeny based on Ref.^93^.

*B. terrestris* belongs to the type subgenus *Bombus s.s.*^31^, a lineage that has long been contentious regarding species status of many members of the complex. Part of this complexity has resulted from the color polymorphisms exhibited by the lineage, which have led to false inferences regarding species boundaries^32,33^. One of the most variable regions in coloration across the subgenus is the regional module involving the dorsum of the third mesosomal (metathoracic) segment (scutellum), the first metasomal tergite, and the posterior thoracic pleuron. This region transitions between black and yellow with intermediacy for some species, and can vary by species, region, and sex across this subgenus (Fig. 1D). Color variation in these segments is also found more broadly across the bumble bees^16,17^. *B. terrestris* also exhibits color variation, with nine regional color forms recognized as distinct subspecies^34,35^. However, in all of these forms both males and females are characteristic black in the abovementioned module (Fig. 1). During rearing of inbred lines of *B. terrestris dalmatinus,* obtained from Israeli stock populations, a bee containing yellow in the aforementioned segments that are typically black was produced and subsequently inbred to develop a yellow mutant line. This fortuitous mutant enables assessment of the genetic basis of this trait using laboratory crosses.

Recent studies^36,37^ have identified black and ferruginous coloration in bumble bees to be imparted by melanin pigments (black eumelanin vs. ferruginous pheomelanin)^36^, while yellow pigments likely involve both a pheomelanin^37^ and a novel pterin-like pigment that occurs in yellow setae across bumble bee species^38^. Given the shift in melanin composition, candidate genes from the *Drosophila* melanin pathway^39^ could be implicated in this color variation. A core set of key developmental transcription factors (e.g., *bric à brac (bab)*, *wingless (wg)*, *Distal-less (Dll)*, *Abd-B*, *engrailed (en)*, *doublesex (dsx)*) has been linked to segmental variation in *Drosophila* pigmentation^39^, thus upstream developmental modulators could also be implicated in this case, especially given the segment-specific location of the phenotype. Tian et al.^12^ for example, found that segment-specific variation involving red or black in mid-abdominal bumble bee segments was driven by cis-regulatory homeotic shifts in the posterior segmental *Hox* gene *Abd-B*. In *Drosophila*, morphological characteristics of the abovementioned set of segments are determined by the *Hox* gene, Ultrabithorax *(Ubx)*^40^. The localization of *Ubx* could explain why this region operates as a module. There are many additional developmental players that have been implicated in spatially preprogramming color pattern elements which could hypothetically program segmental patterning in these bees^41^. Unravelling the SNPs/genes driving this mutant yellow coloration can expand our understanding of genetic targets for driving pigmentation and segmental differences, and potentially reveal underlying genetic processes by which the inheritance of this black to yellow shift occurred within the entire *Bombus s.s.* and broader bumble bee lineage.

In our current research study, we aim to unravel the genomic basis of mutant yellow coloration in *B. terrestris*. To achieve this goal, we utilize a combination of genome-wide reduced-representation approach (RAD-Seq) and whole-genome resequencing data on crossed offspring of both color phenotypes, which we analyze using genotype–phenotype association analyses to identify the genomic region associated with the black to yellow transition. We then investigate the potential function of the implicated mutation by comparing the predicted protein structure elements between wildtype and mutant protein sequences. Finally, we determine the evolutionary history and degree of sequence conservation of the identified genomic region in closely related species and across hymenopterans with publicly available genome sequences to better understand its potential role beyond this lab-generated *B. terrestris* mutation. This study reveals a key developmental gene involved in pigmentation pathways in arthropods which could serve as a candidate gene for further research on extensive color pattern diversity in bumble bees.

## Results

### Phenotypic assessment

The mutant yellow and wildtype black color forms included in this study are outlined in Fig. 1a–c. Although changes in color are most notable in the metathoracic and first metasomal segment, mutant forms have increased yellow hairs in several body regions. On the head of the mutant form there is an approximately 50% mix of yellow setae mixed with black setae on the face and posterior to the eyes. The wildtype bees have nearly all black hairs in these regions and no yellow setae behind the compound eyes. The top of the head (vertex) is mostly yellow in the mutant form and mostly black in the wildtype. The mutant form is yellow on the metathoracic segment, mixed yellow and black on the second (mesothoracic) segment, yellow on the pleuron, and yellow in the ventral region. The wildtype is fully black on the second and third thoracic segments and nearly all black in the ventral thorax and thoracic pleuron. On the metasoma, the first segment is yellow in the mutant and black in the wildtype. The third segment is mostly black with a thin yellow line at the distal boundary in the wildtype but this distal yellow line is thicker and runs up the lateral parts of the segment in the mutant form. The venter (sternites) has substantially more yellow hairs in the mutant form. The mutant form also has more yellow hairs on the femur and parts of the tibia, whereas the wildtype has only black hairs in these parts. No morphological effects outside of setal traits were observed.

The yellow trait was deemed to be recessive and likely a result of a single allele, as heterozygous females are wildtype and her male offspring are 50% yellow form and 50% wildtype. While the initial haploid male mutant contained mostly pure yellow coloration in the respective segments, subsequent crosses reinforced the phenotype, making the yellow in the respective segments more pure (less intermediate/admixed with black), thus additional allelic variants may have minor effects. Accompanying this yellow mutation was a shift in female behavior. Yellow females showed reduced interest in mating with males of any phenotype or line, and low overall mating frequency (~ 20% compared to ~ 80% in wildtype).

### Gene localization: RAD-Seq and GWAS

**Figure 2:**
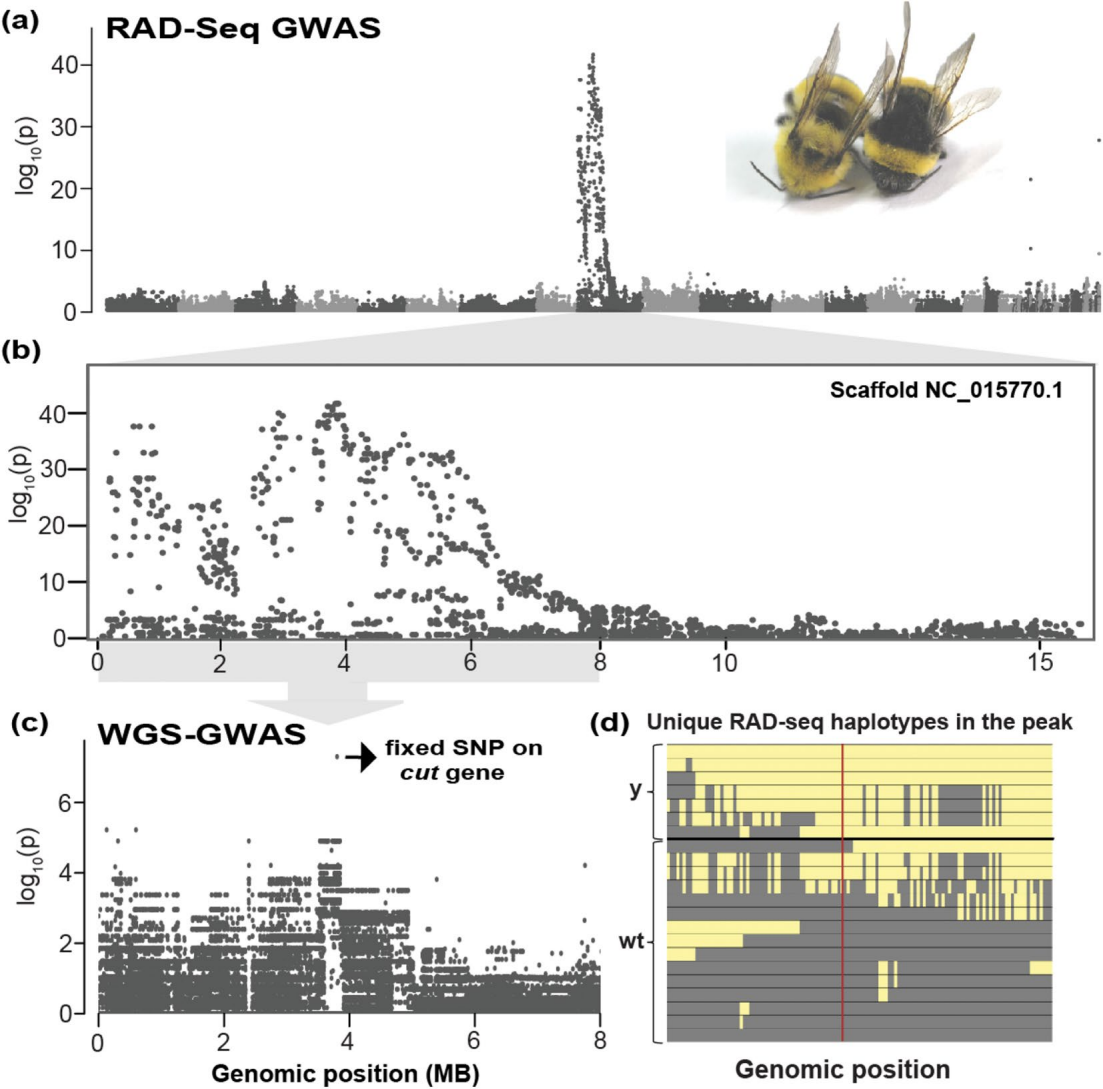
Identifying the color locus through a combination of WGS and RAD-Seq approach (**a**) A genome-wide Manhattan plot using RAD-Seq data reveals a single broad peak of association in scaffold NC_015770.1. Scaffolds represented in different grayscale shades. (**b**) Zoomed view of the Manhattan plot for scaffold NC_015770.1 (**c**) A Manhattan plot using whole-genome sequencing data of the peak region from RAD-Seq. A single fixed SNP on *cut* gene is highlighted. (**d**) A diagram of the unique haplotypes observed across RAD-Seq data in a ~ 3 MB (Genomic locations 2015753-5005430 on scaffold NC_015770.1) region of highest association used to select seven genetically distinct individuals for genomic sequencing of each color form. This is based on actual haplotypes of sampled individuals, focusing on a subset of SNPs that varied primarily between yellow mutant and wildtype individuals. SNPs are colored the color of the variant with the highest frequency of each allele (Yellow [y] for the mutant and gray for the wildtype [wt]). The red line indicates the position in the GWAS of the actual fixed SNP (not sequenced in RAD-Seq data). The filtered SNP dataset is deposited in Dryad digital repository [10.5061/dryad.wstqjq2kr].

Utilizing a combination of RAD-Seq and whole genome sequencing (WGS) on F2 offspring from crossed wildtype and mutant color forms, we narrowed the allele involved in the mutant yellow phenotype to a single nucleotide. First, 57,712 SNPs identified from the 90 RAD-Seq samples, revealed a single broad genomic region (~ 6 Mb) on chromosomal scaffold NC_015770.1 of *B. terrestris* (Bter_1.0, GCA_000214255.1)^28^ that is highly associated with this color trait change (black vs. yellow) (Fig. 2a,b). As RAD-Seq provides a reduced representation of genomic variation, unsurprisingly, this failed to yield fixed SNP differences. We thus utilized whole-genome sequencing (Fig. 2c,d) on 7 individuals of each phenotype that represent unique haplotypes from RAD-Seq data to target SNPs in the implicated interval from RAD-seq. GWAS (Genome-wide Association Study) analysis within this narrowed scaffold (NC_015770.1) revealed a single fixed point mutation (C (mutant), G (wildtype); the genomic position 3802598) which falls at the top of the association peak from the RAD-Seq data. This SNP was determined to fall in the exonic sequences of the homeobox transcription factor *cut.* Our genotyping of this color locus across individuals utilized in the RAD-Seq analysis revealed a complete fixation of this SNP between wildtype and yellow mutants (data available in Dryad [10.5061/dryad.wstqjq2kr]). It is unlikely that any other potential variants were missed in this analysis as at least 95.87% bases were covered at 1X coverage for all samples, a negligible fraction (0.1–3.21)% of regions in a particular sample were discarded for not meeting the required 3× depth coverage, and we allowed up to 25% missing data across all samples to assess fixation for a particular variant. We did not identify any fixed indels in the aforementioned 6 Mb region in subsequent indel analysis.

### SNP conservation and origin

All comparisons to other taxa outside of our samples revealed that they possess the wildtype allele in this position, thus supporting that this mutant is lab generated. Analysis of a publicly available whole-genome resequencing dataset of wildtype populations of *B. terrestris*^29^ revealed only the variant present in our wildtype individuals (n = 22; SNP dataset provided in Dryad digital repository [10.5061/dryad.wstqjq2kr). Genotyping of *cut* DNA sequences of multiple (n = 7) members of closely related species belonging to the *Bombus s.s.* subgenus (Supplementary Table 1) revealed that all of these species also possess the “wildtype” allele, regardless of their yellow and black phenotypes (GenBank accession numbers: MW816640-MW816646). A BLAST (blastn) search across all Hymenoptera (NCBI:txid7399; n = 143) and analysis of aligned *cut* protein orthologs (n = 40) obtained from OrthoDB database^42^ (protein alignment file is provided in Dryad digital repository [10.5061/dryad.wstqjq2kr]) also revealed no SNP or amino acid variation respectively, as all of them have the *B. terrestris* wildtype allele.

### Exploring the function of the implicated mutation

There are nine exons of the *cut* gene (total CDS length, 4806 bp) based on the predicted annotation (NCBI mRNA Reference Sequence: XM_012311834.2). The identified SNP (Wildtype(G) vs. Yellow mutant (C)) is on the fourth nucleotide of Exon 2 of *B. terrestris cut* mRNA, and induces a non-synonymous mutation (Wildtype (Alanine); Yellow mutant (Proline)) on the 38th codon position in the 1601 aa long protein (NCBI Reference Sequence XP_012167224.1). Our sequencing of *cut* cDNA (GenBank accession numbers: MW816647-MW816650, alignment available on Dryad [10.5061/dryad.wstqjq2kr]) from wildtype and yellow mutant adult samples revealed that they both contained the SNP in their RNA, confirming they are part of the protein as expected based on automated annotations. This gene is not known to have natural transcript splice variants in *B. terrestris* according to its latest annotation, however, in other *Bombus* species present in NCBI and other members of Hymenoptera and Arthropoda, multiple splice variants, including at this boundary, are inferred. Amplified transcripts did not have variation in splicing and did not show signs of residual peaks in chromatograms at the splice boundary that would suggest partial production of alternative transcripts. However, as PCR could miss large insertions and rare products, more thorough transcriptomic analysis would be needed to test whether splice variants are involved.

### Protein Structure and conserved domain structure and the role of mutation

**Figure 3:**
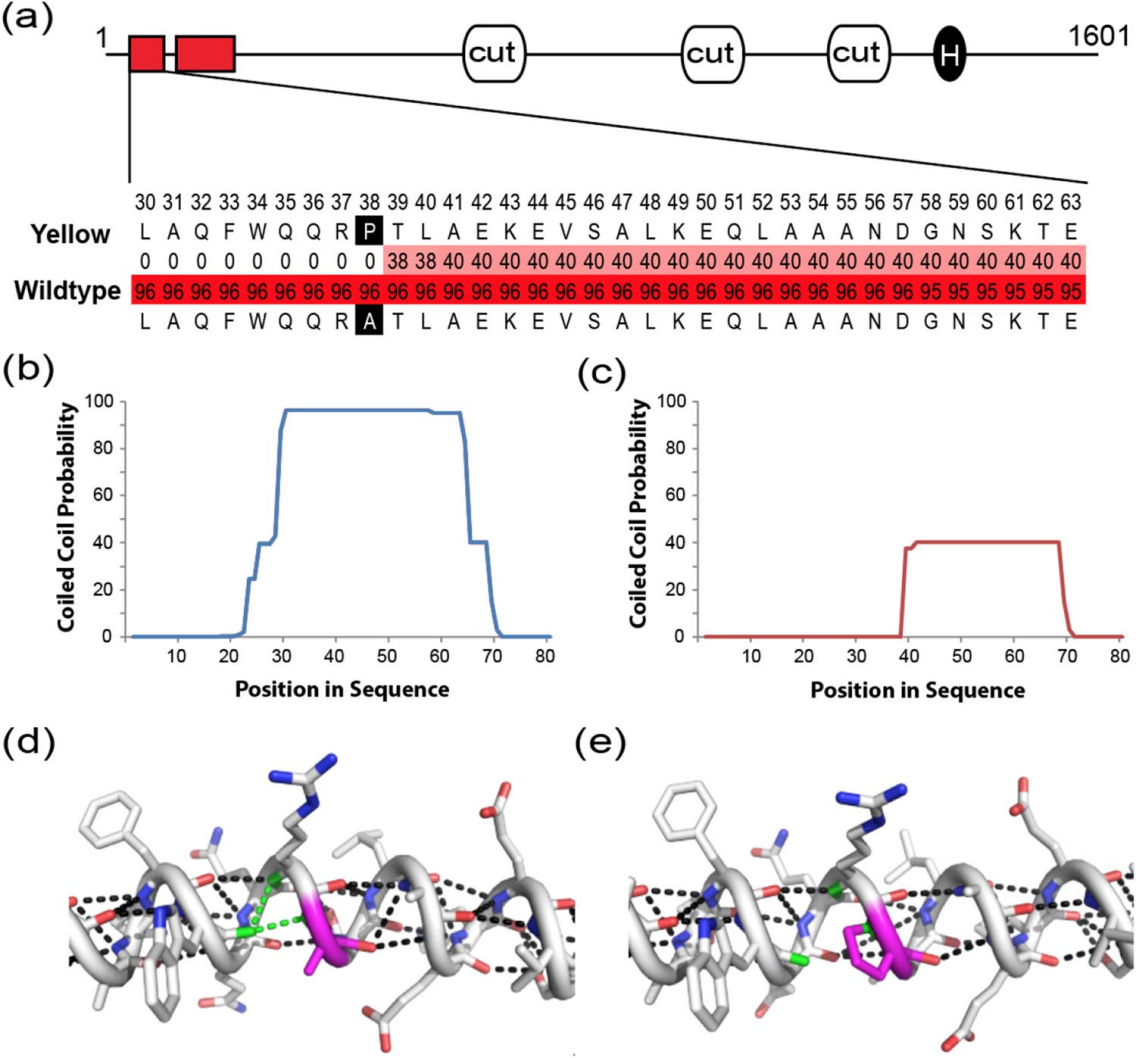
Predicting the structural differences between wildtype and yellow mutant *cut* proteins. (**a**) The *cut* protein sequence includes 3 *cut* domains (grey boxes) and a homeobox domain (light grey circle). Coiled-coil regions (MARCOIL probability > 90) are indicated by red squares. MARCOIL probability list per residue for the wildtype (bottom) and yellow mutant (top) sequence (residues 30–63, labelled above) are highlighted from white to red in color scale from 0 to 100. MARCOIL probability plots for the N-terminal sequence (residues 1–80) of (**b**) wildtype and (**c**) Yellow mutant variant. (**d**/**e**) The *cut* coiled-coil (predicted from residues 20–55) is depicted in cartoon tube from the N-terminus (left) to the C-terminus (right), with residues in stick and colored according to atom type: carbon (white), nitrogen (blue) and oxygen (red); the wildtype sequence (**d**) can form helix stabilizing backbone hydrogen bonds (black dots) surrounding Ala38. Hydrogen bonds form between the Ala38 backbone nitrogen (and the i-1 Arg residue backbone nitrogen) and the i-4 Trp residue backbone carbonyl (green dots). The Ala38Pro mutation removes the ability to form a hydrogen bond between the backbone nitrogen and the i-4 carbonyl (colored green) in the yellow mutant variant (**e**) which destabilizes the helical propensity and can kink the coil.

Figure 3a highlights the domain composition of the *cut* protein sequence, which includes two confidently predicted coiled coils (residues 30–68 and 90–148), followed by three *cut* domains (residues 526–598, 936–1008 and 1170–1239) and a homeobox domain (residues 1294–1347). The Ala38Pro variant maps to the N-terminal coiled coil. The Ala residue present in the wildtype sequence has a helical propensity consistent with the predicted coiled coil secondary structure (Fig. 3a,b). However, mutation of this residue to Pro places a known helix breaking residue in the middle of the coil^43,44^. As a result of this sequence change, the coiled coil prediction becomes less confident (Fig. 3a,c). The helix breaking propensity of the Pro mutation results from an inability of the residue to complete the backbone hydrogen bonding pattern of an a-helix (Fig. 3d,e). As such, coiled-coils infrequently contain Pro residues^45^, and the introduction of a Pro residue into a coiled-coil can lower its helical content and disrupt its oligomeric state by introducing a kink into the helix^46^.

Coiled coils often interact with each other to mediate functional protein interactions. Using physical interactions reported for *D. melanogaster* proteins in BIOGRID^47^, we identified five high throughput interactions (*beag*, *nelf-A*, *nelf-E*, *brd8*, and *Bx42*) with *D. melanogaster cut* that localize to the nucleus and contain predicted coiled-coil sequence regions that could interact with the coil in *cut* (Supplementary Table 2).

## Discussion

Utilizing a combination of reduced-representation and whole-genome sequencing, we identified the color-controlling locus in the yellow mutant phenotype in *B. terrestris* to a single non-synonymous SNP in a homeobox transcription factor, *cut*. *Cut* is a major developmental transcription factor that plays diverse roles in many different cells and tissue types including the brain, sensory organs^48,49^, wing discs, muscle tissues, Malpighian tubules, and reproductive organs^50^, performing as a major selector gene of cell type and fate^51,52^. While the identified functional mutation is confined to the lab mutant and not utilized in natural sister populations, this study adds *cut* to a list of genes that may affect coloration, and which may thus play a role in color patterns in natural bumble bee populations.

Often discovery of genes driving coloration identify pigmentation pathway genes (e.g., Ref.^53–55^). In this case, targeting the genetic basis of a color mutant has led to the discovery of a novel upstream player that can drive aspects of color patterning. It is hypothesized that color patterning along the body is likely dictated by regionally-restricted developmental genes that can then be co-opted to drive spatial differences in pigmentation. For example, complex wing-spot color formation in *Drosophila guttifera* is driven by the co-option of a major developmental gene *wingless* that initially evolved to turn on pigmentation in the wing veins where it was expressed^56^. Using the pre-existing developmental pattern genes to generate novel and localized color-related phenotypes is a recurrent theme in Lepidoptera wing coloration^11,57–60^. Several regionally restricted developmental genes (e.g., *en*, *Omb*, *hh*, *ptc*, *wg*) have been hypothesized to play a major role in generating segment-specific abdominal pigmentation as well^60^.

No clear link between *cut* and body-color pigmentation has been made previously, however, in *Drosophila*, analysis of a *cut* mutant (Dmel\ct^9b2^: FlyBase ID: FBal0028091; first discovered by Hannah^61^ and later catalogued^62^) found that this mutant lead to defective body color, with the fly exhibiting yellowish-tan pigmentation throughout the body. *Cut* may play a role in color patterning through its known interactions with other important developmental regional selector genes, such as *wingless (wg)* and *Notch (N)*^63^. In Lepidoptera, *cut* displays a high-level of co-expression with *wingless* in a spatiotemporal manner, acting as a “molecular cookie-cutter” to determine the complex wing shapes^64^. This *cut*/*wg* boundary determination mechanism in Lepidoptera is evolutionarily derived as it is different from the mechanisms used in *Drosophila* and other holometabolous arthropods, and it may have facilitated the evolution of the astounding wing shape diversity in Lepidoptera^64^.

A long-standing tenet in evolutionary developmental genetics is whether the evolutionary forces are more likely to act on cis-regulatory modules (CRMs) or trans-regulatory (protein-coding) factors. The classic school of thought in evolutionary developmental genetics has emphasized the pre-eminence of cis-regulatory element mutation^65,66^ in generating novel morphological phenotypes. Under this line of thinking, a protein-coding mutation at one of the most important developmental transcription factors should have widespread effects across multiple organs and systems as it is likely to have “deleterious” consequences. Many studies investigating the genetic variation of coloration have revealed the frequent targeting of cis-regulatory elements in highly pleiotropic genes (e.g., Ref.^39,67^) and protein-coding mutations in non-pleiotropic pigmentation genes (e.g., Ref.^53–55^). The fitness consequences of *cut* protein mutation could potentially be substantial, as in *Drosophila* many documented *cut* mutants have displayed lethal or semi-lethal effects^68^. So, how does an apparently deleterious mutation on the protein-coding region of a highly pleiotropic gene such as *cut* have such limited phenotypic effects? In this case this novel mutation (Ala38Pro) occurs outside the characterized homeobox and *cut* domains. It affects protein structure instead through loss of a coiled-coiled structure near its N-terminus (Fig. 3a). Coiled-coiled structures have been speculated to play important roles in protein–protein interactions^69,70^. For example, in *cux1* (cut-like homeobox1) proteins (a gene family in the *cut* superclass and includes *cut* genes in Arthropods) the gain of a coiled-coiled structure in N-terminal region by alternative splicing^69^ results in alternative localization of the protein to Golgi bodies where they act as a transport protein^71^. In the present case, it can be assumed that this mutation has little impact on essential developmental functions of the genes, and likely does not alter all of its roles, as it is not lethal for *B. terrestris* mutant types and has limited phenotypic effect. This leads one to contemplate whether *trans* effects necessarily exhibit high levels of pleiotropy, as altering specific domains of a protein may only affect some of the functions of a protein, for example those where specific proteins are present to interact with, thus generating the more localized tissue-specific effects that typically characterize cis-regulatory modifications. Indeed, a growing body of evidence in recent years demonstrates the importance of protein-coding mutations, which can act in a similar fashion as cis-regulatory mutations to generate modularity in gene regulation given that not all domains are functional in all contexts^72,73^. Another possibility is that the localized effect of the *cut* gene is influenced by the levels of *cut* expression in implicated segments. For example, if the *cut* gene expression in that specific segment is close to the threshold levels needed to invoke specific responses or interaction, modifying a protein structure outside its characteristic protein domains can reduce its functionality and could result in loss of interaction or function at the protein level.

While the consequences of this mutation appear not to be as wide-ranging as the likely function of the *cut* gene, this mutation does have multiple effects on these bees. While the whole body is not yellow, the effects occur in parts of the head, thorax, and upper abdomen. Furthermore, *cut* is known to play roles in reproductive success (e.g., sterility, semi-fertility) in both males and females of *Drosophila,* and to have effects on neurobiology. These functions may also be altered in the yellow mutant *B. terrestris*, as we have observed reduced reproductive success in the mutant, with queens exhibiting worker-like behavior and being less inclined to mate and produce female progeny. This suggests some pleiotropy of this mutation, but also highlights a potential role of *cut* in driving caste-specific behaviors. A link between *cut* and caste specificity has been found in honeybees, where *cut*-like transcripts are downregulated in functionally sterile worker bees (which have only a few ovarioles) compared to its fertile and ovariole-rich queen counterparts^74^. Considering the negative fitness consequences for reproductive success, this particular protein-coding mutation would likely be selected against in nature.

Sanger sequencing of sister species revealed the implicated allele to be novel to this *B. terrestris* lab mutant, as it does not occur in similar phenotypes of closely related *Bombus s.s.* and is not known to occur in any other hymenopteran species. While this SNP is not a result of sorting of ancestral recessive alleles, it is possible that other sequence variants of *cut* may drive similar phenotypic variation in natural bumble bees. Targeting *cut* in cis- may be particularly effective at enabling color variation without pleiotropic consequences.

Through performing laboratory crosses in an emerging model bee, and utilizing a combination of large sample size RAD-Seq and low sample size whole genome sequencing, we have identified a new locus involved in insect color patterning that could also be involved in caste-specific fertility in bumble bees. This novel mutation reveals that protein-coding mutations in major developmental genes can have locally restricted effects. Future genomic research on natural yellow or black color variants in the *Bombus s.s.* lineage is needed to reveal whether this gene may be implicated in this color phenotype in nature, even if this particular mutation is not involved. Increasingly employed genome editing in Hymenoptera (e.g., Ref.^75,76^) as well as differential gene expression studies could be applied to better understand the role of *cut* and this particular mutation in bumble bees.

## Material and methods

### Sampled specimens and phenotypes

Initially a male (*B. terrestris dalmatinus*) with a yellow mutant color form containing yellow in the third thoracic segment, first abdominal segment, and posterior pleuron was generated from wildtype parents. Offspring from this individual were then crossed with wildtype *B. terrestris dalmatinus* and inbred across ~ 18 generations to make a yellow mutant line. The yellow trait is recessive, with heterozygous females having wildtype coloration and her male offspring exhibiting both color forms. Yellow females showed reduced interest in mating with males of any phenotype or line (see Results), which resulted in reduced reproductive output of females and reproductive gynes. A few were produced, however, to maintain the line prior to experimentation, but not enough to maintain it for a longer term, as the line no longer exists. Selected specimens were examined for morphological differences across the body (e.g., wings, facial features, genitalia), including documentation of details of color pattern. Specimens were not examined internally.

Sixty wildtype *B. terrestris* queens from three colonies were placed in an arena and crossed with multiple (~ 70) males from three colonies of the inbred yellow mutant line *B. terrestris* in a commercial bumble bee rearing facility (BizBee, EinYahav, Israel). Ten queens successfully generated offspring, from which eighty hybrid workers (F1) were generated. These workers were divided into four mini-colony worker groups (workers will lay male eggs when together in mini-groups, although not all individuals will lay) and allowed to generate male offspring (F2). Resulting F2 males exhibiting both wild-type and mutant color forms were selected for subsequent DNA sequencing. Given the pooled nature of the design, parentage could not be assessed, but should represent most of the ten original crosses.

### DNA extraction and Sequencing for RAD-Seq

Thoracic tissue from 90 haploid males (including 44 wildtype and 46 yellow mutant individuals), were extracted using a Qiagen DNeasy Blood and Tissue Kit with RNaseA treatment. Samples were extracted into a 250 µl extraction buffer, dried using a SpeedVac, and re-eluted in 50 µl water. Extracted samples were quantified using the Qubit BR dsRNA kit to ensure at least 250 ng of DNA, and were assessed on a 1% agarose gel to ensure high-quality genomic DNA. Samples were prepared for RAD-Seq using protocols following Ref.^77,78^. This involved digesting 250 ng of DNA per sample in a 50 µl reaction with PstI-HF (NEB) for 1 h at 37 °C, followed by 20 min at 80 °C. Samples were divided into six sets (libraries) of 16, in each of which 16 different P1 barcoded adaptors were ligated using T4 DNA ligase with incubation for 22 °C for 1 h, 65 °C for 10 min, and 30 min at room temperature. Each library was pooled and sheared to 300–700 bp using a Covaris S2 sonicator. Gel-based size selection of 300–700 bp fragments was then performed and samples purified using a Nucleospin Gel and PCR clean-up kit. Samples were end-repaired with the Quick Blunting Kit (NEB) and purified with Agencourt AMPure XP (Beckman Coulter) magnetic beads. This was followed by addition of dATP overhangs added with the Klenow exo (NEB), purification with AMpure XP beads, ligation of P2 adaptors, and additional purification with AMpure XP beads. Samples were then PCR amplified (98 °C for 30 s, 16 cycles of 98 °C for 10 s and 72 °C for 1 min, 72 °C for 5 min) using Phusion High-Fidelity Master Mix with specific P2 Sanger indexing primers for each of the six pools. This process enabled the unique barcoding of all 9 samples. RAD-tag short read (2*150 bp, paired-end) libraries were sequenced using an Illumina HiSeq 2500 in Pennsylvania State University Genomics Core Facility (University Park, Pennsylvania, USA).

### DNA extraction and sequencing for whole-genome analysis

We selected a subset of samples for the whole-genome resequencing approach (both wildtype (n = 7) and yellow mutant (n = 7)) from the specimens used for RAD-Seq. To optimize the potential to find fixed sites within the association peak, we identified unique RAD-Seq haplotypes within the peak of association and selected individuals which maximally represented the range of differences in RAD-Seq haplotypes (Fig. 2d). Genomic DNA extraction was conducted from thoracic tissues of haploid males using either Qiagen DNeasy or E.Z.N.A. (Omega Bio-tek) kit followed up by RNaseA treatment. 150 bp paired-end sequencing libraries were prepared using Illumina TruSeq DNA Nano kits for whole-genome re-sequencing approach implemented in Illumina HiSeq 2500 sequencer (5–10 individuals per lane aimed at ~ 30X per sample coverage) in Pennsylvania State University Genomics Core Facility (University Park, Pennsylvania, USA).

### Analysis of RAD-Seq dataset

RAD-Seq analysis was completed using Stacks 2 software^79^ following the recommendations of a published RAD-Seq analysis protocol^80^. First, the raw paired-end sequencing reads for six paired-end libraries were filtered and demultiplexed to generate individual-sample level sequence dataset using process_radtags unit of Stacks 2 software. Reads from individual samples (n = 90, 44 wildtype and 46 yellow individuals) were aligned to the published *B. terrestris* genome assembly (Bter_1.0, GCA_000214255.1)^28^ using BWA aligner v. 0.7.17^81^ and the bwa-mem algorithm with default parameters. After that, we ran pstacks, “cstacks”, “sstacks” and “gstacks” units of Stacks 2 as recommended by^80^ and after applying a stringent filtering criteria (-p 2 -r 0.75; SNP must be present in both yellow and wildtype populations and genotyped in at least 75% of samples) generated a final SNP dataset of 57,712 SNPs using the “populations” unit of Stacks 2 that was ultimately used in genotype–phenotype association analysis. To test the association between case–control phenotypes (wildtype and yellow mutants) with the filtered SNP dataset, we performed a genotype–phenotype association testing utilizing Fisher’s exact test implemented in PLINK v.1.9^82^. Raw RAD-Seq reads for individual samples are available under NCBI BioProject PRJNA716745.

### Analysis of whole-genome resequencing dataset

Whole-genome resequencing data of wildtype (n = 7) and yellow mutant (n = 7) samples were analyzed using a previously implemented bioinformatic pipeline^12^. In brief, we applied appropriate adapter trimming (ILLUMINACLIP:adapters.fa:2:30:5), removal of low-quality bases (SLIDINGWINDOW:4:30 LEADING:3 TRAILING:3) and short-length (MINLEN:36) sequences using Trimmomatic v. 0.38^83^. The published *B. terrestris* genome assembly (Bter_1.0, GCA_000214255.1)^28^ was used as a reference to align the trimmed reads using BWA aligner v. 0.7.17^81^ in bwa-mem mode. Post-processing of aligned reads was implemented in SAMtools v. 1.8^84^ and Picard tools v. 1.119. We ran GATK v. 3.6^85^ in UnifiedGenotyper mode for multi-sample (n = 14) variant calling using a haploidy-specific parameter (-ploidy 1 -glm SNP -stand_call_conf 25.0). Variant quality filtering was conducted in VCFtools v. 0.1.15^86^ using specific parameters (—max-missing 0.75—minDP 3—minQ 30—min-alleles 2—max-alleles 2), retaining high-quality biallelic SNPs and allowing no more than 25% missing data for any SNP position. The final genome-wide SNP dataset included 14,70,101 SNPs. Utilizing this filtered SNP dataset, we implemented Fisher’s exact test in PLINK 1.9^82^ to run a case–control (wildtype vs. yellow mutant phenotype) genotype–phenotype association analysis. To investigate the possible involvement of indels, an additional run of variant calling was conducted where both SNP and indels were called from the whole-genome sequencing data and GWAS was implemented using the aforementioned methods. Summary statistics of analyzed genomic samples are available in Supplementary Table 3 and raw genome sequencing reads are available under NCBI BioProject PRJNA716745.

### SNP validation using sanger sequencing

To genotype the identified fixed SNP from the genotype–phenotype association analysis (see results) across specimens used in RAD-Seq analysis, genomic DNA was extracted from 83 individuals (42 wildtype and 41 yellow mutants) using thoracic tissue and standard protocols of a Qiagen DNeasy Kit. Primers were designed to amplify the candidate SNP-harboring region (SNP position 3,802,598 on NC_015770.1 identified from RAD-Seq and WGS analysis; Bter_CM1177_3802598_L 5′-CCTCTTTGTCCTTCGCTTGC-3′, Bter_CM1177_3802598_R 5′-CCAGCAAGATTCGCGAAATAGT-3′) and were amplified through PCR (94 °C 2 min, 35 cycles of [94 °C 30 s, 51 °C 30 s, 72 °C 90 s], 72 °C 10 min; 15 µl reaction with 0.3 µl 10 uM primers, 5.9 µl water, 1 µl DNA, and 7.5 µl HotStart Taq Mastermix (NEB)), purified with ExoSap-IT or a Qiagen MinElute PCR Purification Kit, and Sanger sequenced at the Pennsylvania State University Genomics Core Facility (University Park, Pennsylvania, USA). Manual inspection, trimming, and alignment of Sanger sequence data (chromatograms) was conducted in Geneious v. 8.1.9 and the previously identified SNP was manually called.

### SNP comparison to other bumble bees and Hymenoptera

To add additional data toward understanding the fixation of the identified SNP by phenotype (SNP position 3,802,598 on NC_015770.1), we downloaded whole-genome sequencing data from 22 wildtype *B. terrestris* individuals available on NCBI (BioProject accession ID: PRJNA326162, sample names from I-D1 to I-D22)^29^. Read trimming, alignment, alignment post-processing, multi-sample (n = 22) variant calling, and variant filtering followed the bioinformatic pipeline for genomic data used above. The resulting SNP dataset (n = 10,79,814) was visualized in IGV v. 2.3.86^87^ to identify whether the SNP remained fixed in the color locus considering their wildtype phenotype.

To test whether the identified color locus is implicated in wild populations of *B. terrestris* relatives, we included seven additional members of closely related species belonging to subgenus *Bombus s.s.* that vary in whether the above-mentioned segments are yellow or black (*B. patagiatus* (Yellow, Worker), *B. lucorum* (Black, Worker), *B. hypocrita* (Yellow, Male), *B. terricola* (Mostly Black, Queen), *B. sporadicus* (Yellow, Worker), *B. cryptarum s.s.* (Black, Worker), and *B. cryptarum moderatus* (Black, Worker); Fig. 1d; Supplementary Table 1). Genomic DNA extraction, PCR, PCR purification, sequencing, sequence editing and allele calling followed protocols outlined above for genotyping of the narrowed locus.

To determine the nucleotide and amino-acid conservation of the implicated mutation more broadly across Hymenoptera, we performed multiple local-alignment searches (blastn, tblastn, blastx, blastp and tblastx) using NCBI BLAST web interface and downloaded gene orthologs (n = 40) (Group 1911at7399) of all hymenopterans from OrthoDB catalogue release 10.1^42^. Amino acid sequences of orthologous proteins were aligned in Geneious v. 8.1.9 using MAFFT alignment program applying E-INS-i algorithm (default parameters: Scoring matrix: BLOSUM62, Gap Open Penalty 1.53, offset value 0) and the amino acid sequence conservation was visually compared.

### Gene annotation

To determine whether the SNP was located in a protein or cis-regulatory region, the SNP was compared against gene annotations available for *B. terrestris* on Hymenoptera Genome Database^88^ where annotations have been made using automated approaches facilitated by transcriptome data, and the implicated region was further investigated through BLAST searches to NCBI database to manually check this annotation. Although this supported the implicated SNP belonging to a protein-coding region, it suggested that the SNP was just three bases away from an intron–exon boundary. To check whether the SNP was contained in the final transcript and assess the potential for alternative splicing, we sequenced transcripts including the implicated region extracted from the head and abdominal tissues from one wildtype and one yellow mutant form. RNA extraction and DNA removal were conducted with the Direct-zol RNA Miniprep Plus kit following homogenization of tissue in 400 µl Trizol using 4 metal beads in a 2 ml tube in an Omni Bead Ruptor for 35 s. cDNA synthesis was conducted using a 15 µl reduced volume reaction (includes 10.7 µl of 500 ng RNA) but otherwise performed using standard protocols for the High Capacity cDNA Reverse Transcription Kit. Designed primers amplified a 230 bp fragment that spanned across three exons and two introns, including the intron nearest the implicated mutation (intron 1) (HHCutF_Exon1 5′ ACATTCAGGCCATGCAGTC 3′, HHCutR_Exon3 5′ GCTCTGCTGTAACCTGGACA 3′). PCR amplification was conducted using NEB 2X Hot Start Mastermix in a 15 µl reaction with the following conditions: 95 °C for 2 min, 30 cycles of 95 °C for 30 s, 52 °C for 30 s, 72 °C for 1 min, 5 min at 72 °C. A long-run high-density gel electrophoresis (2% agarose gel) was performed which confirmed just a single band for each PCR. Sanger sequencing of the PCR products (with HHCutF_Exon1 primer only) were conducted at the Pennsylvania State University Genomics Core Facility (University Park, Pennsylvania, USA). We utilized Geneious v. 8.1.9 for manual inspection, trimming and alignment of Sanger sequence data (chromatograms) against publicly available *B. terrestris cut* cDNA sequences (NCBI Reference Sequence: XM_012311834.2).

### Protein structure and domain analysis

We tested whether the identified mutation alters structural and functional properties of the protein by performing translation analysis, predicting protein–protein interactions and structural domains of the identified protein, and determining whether predicted secondary structure is altered. Protein domains from Pfam^89^ were assessed using default NCBI conserved domain database search, and coiled coils were predicted using the program MARCOIL with a threshold of 90 from the HHPRED server toolkit^90^. Because the identified mutation (Ala38Pro) falls within the first predicted coiled coil region (amino acid residue range 30–63) of the *cut* protein, we repeated MARCOIL prediction using the SNP variant using the N-terminal 80 residues of each sequence. Potential protein interactions for *cut* were investigated using BIOGRID^47^ for *Drosophila melanogaster* orthologs. *D. melanogaster* protein sequences reported to interact with *cut* were submitted to MARCOIL prediction, and sequence ranges of coiled-coils with probability above 90 were reported. *D. melanogaster* ortholog protein localization was reported from UniProtKB^91^ annotations. PubMed identifiers from BIOGRID and *D. melanogaster* ortholog proteins from *B. terrestris* are reported for nuclear proteins with predicted coils that could potentially interact with the N-terminal coil in *cut*. We made structure models of the wildtype and yellow mutant coiled coil sequence (residues 20–55) using a helix identified from top template structure 6RW9_A (residues 2143–2178) with HHPRED.

## Supplementary Information

Supplementary Information.

## Data Availability

Raw sequence reads for specimens used in RAD-Seq and WGS approaches are deposited under NCBI BioProject PRJNA716745. cDNA sequences of *cut* sequences collected from head and abdominal tissues from wildtype and yellow mutant for testing alternative splicing in *B. terrestris* and cDNA sequences obtained for genotyping the color locus in *Bombus s.s.* are deposited in NCBI GenBank (Accession Nos.: MW816638-MW816650). Datasets are provided in the Dryad digital repository (10.5061/dryad.wstqjq2kr) including final SNP datasets from publicly available wildtype *B. terrestris* sequencing data and in-house RAD-Seq and WGS based GWAS approaches along with associated population (phenotype) assignment files, MAFFT alignment file (nexus format) from protein homologs from OrthoDB database, manually edited alignment files (nexus format) from SNP validation in *B. terrestris* RAD-Seq samples.
