# Supplementary material for: A combined RAD-Seq and WGS approach reveals the genomic basis of yellow color variation in bumble bee Bombus terrestris
Source: Sci Rep. 2021 Apr 12;11:7996. doi: 10.1038/s41598-021-87194-y (PMC8042027; doi:10.1038/s41598-021-87194-y)
Supplement: Supplementary file 1 — Supplementary Information. [file 41598_2021_87194_MOESM1_ESM.docx]

**Supplemental Information**

**Supplementary Table 1: Sample information for closely related species of *Bombus s.s.* Specimens are the same as those used in other studies, includes ID and reference from those studies.**

| Molecular ID | *Bombus* sp. | Caste/Sex | Color T3/M1 | Locality | Note |
| --- | --- | --- | --- | --- | --- |
| BCP001 | *patagiatus* | Worker | Yellow | China: Sichuan, Tibetan Plateau, 3533m, 10-50km S. Hongyuan; 4-VIII-2002 |  |
| BCP003 | *lucorum* | Worker | Black | Switzerland: St. Gotthard's Pass; 27-VII-1999 |  |
| BCP005 | *sporadicus* | Worker | Yellow | Sweden: Abisko; 25-VII-1999 | SC193^93^ |
| BCP006 | *cryptarum moderatus* | Worker | Black | USA: Alaska | CP016A^17^ |
| BCP007 | *cryptarum armeniacus* | Worker | Black | Turkey: Erzingan, Yeniyol-Ahmitli, 2120m; 5-VIII-2002 |  |
| BCP009 | *hypocrita* | Male | Yellow | Russia: Primorskiy Krai, Muravjov-Amurskii Peninsula; 25-VIII-2002 | SC168^93^ |
| Btrc042 | *terricola* | Queen | Mostly Black | USA: Pennsylvania, State College; 24-IV-2014 |  |

**Supplementary Table 2 :BIOGRID interactions for *D. melanogaster* *cut* protein**

| NCBI Gene ID | Gene name | BIOGRID throughput | Coiled- coiled regions (MARCOIL prediction probability threshold >90) | UniProtKB Annotation Localization Information | PubMed ID | *B. terrestris* ortholog ID |
| --- | --- | --- | --- | --- | --- | --- |
| CG8597 | *lark* | Low | No | Nucleus, Cytoplasm | 27662615 | NA |
| CG18005 | *beag* | High | 186-227 | Nucleus, spliceosome | 25242320 | XP_012175085 |
| CG5874 | *nelf-A* | High | 277-304 | Nucleus, Chromosome | 25242320 | XP_003401362 |
| CG14514 | *brd8* | High | 112-173 | Nucleus | 25242320 | XP_012172412 |
| CG5994 | *nelf-E* | High | 8-36 | Nucleus, Chromosome | 25242320 | XP_003396851 |
| CG8264 | *Bx42* | High | 296-330 | Nucleus | 25242320 | XP_003397263 |
| CG16932 | *eps-15* | High | 423-552 | Extracellular | NA | NA |
| CG9638 | *ada2b* | High | No | NA | NA | NA |
| CG18297 | *cdk2ap1* | High | No | NA | NA | NA |
| CG17252 | *bcl7-like* | High | No | NA | NA | NA |
| CG1520 | *wasp* | High | No | NA | NA | NA |
| CG6521 | *stam* | High | No | NA | NA | NA |
| CG11482 | *mlh1* | High | No | NA | NA | NA |

**Supplementary Table 3: Summary Statistics for WGS Samples**

| Specimen Name | Phenotype | NCBI BioSample ID | Number of pre-QC read pairs (M) | Number of post-QC read pairs (M) | Sequencing Depth  (Average Coverage) | % reads aligned to Reference |
| --- | --- | --- | --- | --- | --- | --- |
| BterW_0085 | Wildtype | SAMN18450444 | 39.94 | 32.77 | 27.93 | 97.67% |
| BterW_0072 | Wildtype | SAMN18450438 | 29.54 | 21.88 | 20.15 | 97.15% |
| BterW_0100 | Wildtype | SAMN18450452 | 20.79 | 18.03 | 16.78 | 97.48% |
| BterW_0031 | Wildtype | SAMN18450423 | 26.82 | 20.61 | 18.43 | 97.88% |
| BterW_0049 | Wildtype | SAMN18450432 | 26.58 | 20.37 | 18.12 | 97.84% |
| BterW_0009 | Wildtype | SAMN18450414 | 24.30 | 17.53 | 17.87 | 97.78% |
| BterW_0011 | Wildtype | SAMN18450415 | 27.55 | 17.70 | 17.13 | 97.79% |
| BterY_0026 | Yellow | SAMN18450476 | 18.75 | 14.75 | 12.79 | 97.51% |
| BterY_0005 | Yellow | SAMN18450458 | 16.21 | 14.97 | 13.89 | 97.27% |
| BterY_0019 | Yellow | SAMN18450470 | 30.66 | 23.90 | 21.52 | 97.17% |
| BterY_0021 | Yellow | SAMN18450471 | 27.79 | 20.88 | 19.09 | 97.87% |
| BterY_0057 | Yellow | SAMN18450493 | 30.43 | 23.99 | 22.50 | 97.76% |
| BterY_0003 | Yellow | SAMN18450456 | 26.82 | 18.82 | 19.02 | 97.79% |
| BterY_0042 | Yellow | SAMN18450483 | 27.24 | 18.61 | 18.59 | 97.76% |
